# Distal Entrapment of Regenerating Peripheral Nerves After a Proximal Injury: A Case Series and Review of the Literature

**DOI:** 10.7759/cureus.50756

**Published:** 2023-12-18

**Authors:** Alexander J Baldwin, Chane Kulenkampff, Dominic M Power

**Affiliations:** 1 Department of Burns and Plastic Surgery, Stoke Mandeville Hospital, Aylesbury, GBR; 2 The Peripheral Nerve Injury Service, Queen Elizabeth Hospital Birmingham, Birmingham, GBR

**Keywords:** nerve decompression, neural regeneration, nerve entrapment, peripheral nerve surgery, peripheral nerve injury

## Abstract

A complication of peripheral nerve injuries, of which there exists limited discourse, is the entrapment of the nerve as it regenerates from the site of injury to its end target, resulting in the arrest of axon regeneration and a consequent reduction of functional recovery. This proof-of-concept paper reports a review of the relevant literature alongside a case series of patients who presented with this phenomenon and who were treated with targeted peripheral nerve decompression.

Three cases were identified prospectively. The baseline function was recorded pre-and post-operatively. Recovery was assessed using various tools, including the Medical Research Council (MRC) motor grading, ten-test sensory testing, Tinel's sign progression, a visual analogue scale (VAS) for pain, and the Impact of Hand Nerve Disorders (I-HaND) patient-reported outcome measure (PROM).

The first case sustained a brachial plexus injury and received decompression at the pronator fascia, carpal tunnel, cubital tunnel, and Guyon’s canal. The second case sustained a sciatic nerve injury and was managed with peroneal and tarsal tunnel decompressions. The final case sustained a suprascapular nerve injury and underwent decompression at the suprascapular ligament. In all these cases, motor function, sensory function, and pain (depending on the nerve's original components) improved following decompression. A literature review revealed seven relevant studies, including four case reports, two cohort studies, and a pre-clinical animal study.

These cases, and those identified in our review of the literature, suggest that targeted decompressive surgery can be an appropriate treatment for patients who display signs of stalled neural regeneration. This study adds to the limited evidence of this phenomenon and highlights the challenges in proving the efficacy of decompressive surgery for this specific complication. This study is limited by the number of cases included, the heterogeneity of nerve injuries presented, and its observational nature. There is a clear need for further research into this phenomenon, and the authors are working towards developing a prospective study that will investigate the indications, value, predictors of success, and practicality of decompression surgery for this complication of peripheral nerve injury.

## Introduction

Peripheral nerve injuries result in substantial morbidity, and recovery is frequently incomplete. Their long-term sequelae can have devastating effects on a patient’s quality of life. Moreover, they can result in huge social and economic burdens for both individuals and the state [1]. These injuries often result from trauma and are more common in the upper limbs [1, 2].

Subsequent entrapment of a recovering nerve as it regenerates from the site of injury to its end target is a phenomenon that has been infrequently described in the medical literature [3-9]. Entrapment of an injured nerve can result in the slowing, or arrest, of axon regeneration, with a consequent reduction in motor and sensory functional recovery [3-9]. Injured nerves are more susceptible to entrapment owing to the distal end of the proximal regenerating segment having a greater volume than an intact, functioning nerve. There is a disruption of axoplasmic flow near the terminal nerve, and the nerve stump has a growth cone containing multiple microtubules for cytoskeleton assembly and projecting filopodia [3-9]. This renders it vulnerable to compression by external structures at common anatomical entrapment points. It has been postulated that the variance of occurrence of this complication may be due to the presence of a subclinical entrapment predating their injury in a subset of patients, otherwise known as a ‘double-crush’ syndrome [5].

In the upper limb, the radial nerve may be compressed at the lateral intermuscular septum of the upper arm, the posterior interosseous nerve at the proximal edge of the supinator muscle, and the superficial radial nerve at the dorsal edge of the brachioradialis tendon in the distal half of the forearm. The ulnar nerve may become entrapped as it negotiates the cubital tunnel or Guyon’s canal. The median nerve may be compressed at the lacertus fibrosus (bicipital aponeurosis), the pronator teres, the flexor digitorum superficialis, the proximal fibrous arch, or the carpal tunnel [1, 2]. Lower limb nerves may be similarly affected, with the common peroneal nerve vulnerable to entrapment at the peroneal tunnel, the superficial peroneal nerve at the distal lateral fascia of the lower leg, and the deep peroneal nerve proximally at the anterior crural septum or distally at the extensor retinaculum of the ankle. The tibial nerve may be liable to compression at the soleus arch in the upper posterior calf or the tarsal tunnel [1, 2].

This proof-of-concept paper reviews the relevant literature and reports a case series of three patients with distal nerve compression following a proximal injury who underwent targeted peripheral nerve decompression.

Materials and methods

A literature search combining variations of the terms "nerve," "decompression," “distal compression/entrapment,” and “proximal injury” was performed on the Medical Literature Analysis and Retrieval System Online (MEDLINE, PubMed), The Cochrane Library, and Google Scholar databases. In addition, reference lists were scrutinised for relevant studies.

A review of three cases with prospectively gathered data chosen to illustrate this phenomenon was undertaken. Potential cases with symptoms suggestive of distal compression were identified in patients attending clinics of a tertiary peripheral nerve injury service. The development of a static Tinel’s point at a known natural compression point with a weak and slowing distal Tinel’s sign was the primary method of identifying patients for further evaluation. Secondary parameters included persistent neuropathic pain and direct compression testing at the site of suspected entrapment, causing exacerbations of pain and paraesthesia. These findings, coupled with poorer-than-expected motor and sensory recovery, were used to define patients for consideration of intervention with targeted peripheral nerve decompression.

The baseline function was recorded pre-operatively and subsequently in the post-operative period. Due to the varied sites and severity of the included injuries and the inability to accurately employ neurophysiology in low-amplitude studies, a number of clinical signs were combined with pain scores, subjective sensory scores, functional assessments, and location-specific patient-reported outcome measures (PROMs) to better evaluate the complex functionality provided by peripheral nerves. Medical Research Council (MRC) motor grading [10], ten-test sensory testing [11], rate of Tinel’s sign progression [12], the differential Tinel’s sign, and a visual analogue scale (VAS) were used depending on the nature of the affected nerve. These were combined in one case with the Impact of Hand Nerve Disorders (I-HaND) tool, a 32-item hand-specific PROM developed to track the recovery of sensory, motor, and mixed peripheral nerves after injury [13].

The progression of Tinel's sign refers to the sensation produced by tapping an injured nerve and is interpreted as demonstrating the presence of regenerating axons, which have immature myelination and lowered thresholds for mechanical stimulation [12]. Nerves are tested from distal to proximal, with the most distal Tinel's point representing the location that axons have regenerated to. The differential Tinel's sign can be used to determine the success of regeneration. A stronger Tinel’s elicitation more proximally than distally may suggest poor repair or a high-grade injury with intraneural scarring and the formation of neuroma in continuity. Stalling of the advancing Tinel's sign may suggest external compression impeding axonal regeneration [14]. The ten-test is used to assess sensation by comparing affected and unaffected limbs and giving this a score out of 10. This test thereby addresses the subjective nature of assessing sensation [11]. The ten-test provides no useful discrimination of function between subjects; however, it is a useful tool for monitoring an individual subject. The VAS can be used to track an individual's neuropathic pain, which is a common feature of peripheral nerve injury but is limited by assuming a linear experience of pain [15]. Despite this, it still serves as a useful tool to track pain and determine if an intervention has been effective. The MRC scale is widely used to examine power, with improvements indicating recovery of motor function [10]. Power is scored on a grade of 0 to five, with grade 0 being no contraction and grade 5 corresponding to normal power [10]. A limitation of this scale is that over 90% of absolute myometric measurements fall in grade 4, covering non-functional to virtually normal readings. In addition, testing a muscle with a single reading provides no information on endurance or fatigue [10, 15].

## Case presentation

Our case series comprises two upper and one lower limb peripheral nerve injuries that were subsequently managed with targeted decompressive surgery following the identification of a static Tinel’s sign and/or a delay in the recovery of function. A summary of these can be seen in Table 1.

**Table 1:** Summary of the cases MRC: Medical Research Council power grading score; VAS: visual analogue scale (0-100, 0 = no pain, 100: severe pain); I-HaND: Impact of Hand Nerve Disorders; CPN: common peroneal nerve; TN: tibial nerve; SPN: superficial peroneal nerve; PL: peroneus longus; EDL: extensor digitorum longus; EHL: extensor hallucis longus; TA: tibialis anterior; DPN: deep peroneal nerve; SPN: superficial peroneal nerve; TN: tibial nerve; SA: shoulder abduction (supraspinatus and deltoid), IS: infraspinatus, TM: teres minor

Case	Age (years), Gender	Injury	Time from injury to surgery (weeks)	Decompression sites	Time from surgery to follow-up (weeks)	Pre-operative MRC grade	Post-operative MRC grade	Pre-operative VAS	Post-operative VAS	Pre-operative ten-test	Post-operative ten-test	Pre-operative I-HaND	Post-operative I-HaND
1	61, Female	Left infraclavicular brachial plexus injury involving all three cords	44	1. Pronator fascia, 2. Carpal tunnel, 3. Cubital tunnel, 4. Guyon’s canal	6	1	2	75	40	7/10	10/10	74	20
2	19, Male	Right sciatic nerve injury with CPN and TN involvement	52	1. Peroneal tunnel, 2. Scar overlying SPN, 3. Tarsal tunnel	6	PL: 3, EDL: 3, EHL: 3, TA: 0	PL: 4, EDL: 4, EHL: 4, TA: 2	60	40	DPN: 0/10, SPN: 6/10, TN: 6/10	DPN: 1/10, SPN: 8/10, TN: 9/10	-	-
3	35, Male	Right suprascapular nerve injury	40	Suprascapular ligament	40	SA: 1, IS: 0, TM: 3	SA: 5, IS: 4, TM: 4	-	-	-	-	-	-

Case one

A 61-year-old female sustained a left infraclavicular brachial plexus injury following a fracture-dislocation injury of the proximal humerus, which was subsequently managed with reverse arthroplasty. At presentation, she had numbness, dry skin affecting the whole hand, and no motor function. These features were indicative of axonopathy affecting all three cords. The loss of sudomotor function demonstrates disruption of the small, unmyelinated autonomic fibres in addition to the loss of the larger, myelinated sensory and motor fibres. Additionally, the patient reported severe neuropathic pain, which is a feature suggestive of axonopathy. Neurophysiology confirmed evidence of widespread, significant active degeneration with no functioning motor axons under voluntary control within the cords of the left brachial plexus.

At four months following her index injury, there was evidence of regeneration with a Tinel’s sign elicited within all three nerves at the level of the elbow. The rate of progression for each nerve was determined at between 2-3 mm per day, with a point of low-grade axonopathic injury assumed to be at, or close to, the level of the coracoid process. At 10 months post-injury, her pain had decreased, and there was recovery of the function of the extrinsic digital flexors and extensors due to early reinnervation of the proximal forearm muscles. However, a persistent and strong Tinel’s sign was elicited over the median nerve at the flexor digitorum superficialis arch and an additional, albeit less prominent, Tinel’s sign at the flexor retinaculum overlying the left carpal tunnel. Similarly, the ulnar nerve elicited a strong Tinel’s sign at the cubital tunnel, and a weaker Tinel’s sign affecting the sensory component was elicited at Guyon’s canal. The patient was offered and consented to decompressive surgery at the pronator tunnel, the carpal tunnel, the cubital tunnel, and Guyon’s canal.

Prior to the surgery, the clinical assessments included an I-HaND score of 74, a VAS of 75 (1-100), MRC grade 1 for the muscles distal to the positive Tinel’s points, and a ten-test of 7/10. At six weeks following surgery, the patient was finding it much easier to move her fingers, and she felt that her sensation had markedly improved. At this point, her outcome measures were an I-HaND score of 20 (a reduction of 54), a VAS of 40 (1-100), MRC grade 2 for the muscles distal to the Tinel's, and a ten-test of 10/10.

Case two

A 19-year-old male motorcyclist was involved in an accident and sustained polytrauma, including a right-sided sciatic nerve injury. His concomitant injuries included an ipsilateral open femoral shaft fracture, a traumatic brain injury with a temporal lobe contusion, a right-sided complex open fracture of his elbow, and a degloving injury to his right lower leg. The patient made an excellent recovery from his traumatic brain injury but continued to experience neuropathic pain in his right foot and marked weakness of ankle dorsiflexion as a result of his sciatic nerve injury. Neurophysiology studies confirmed severe dysfunction of the right common peroneal nerve with some preservation of tibial nerve function.

Examination at four months following the injury revealed considerable motor impairment of the right lower limb, with barely any contraction palpable (MRC grade 1) in the peroneus longus (PL), extensor digitorum longus (EDL), extensor hallucis longus (EHL), tibialis anterior (TA), flexor digitorum longus (FDL), flexor hallucis longus (FHL), and tibialis posterior (TP). There was absent sensation in the distribution of the deep peroneal nerve, reduced sensation in the tibial nerve distribution, and dysaesthesia in the superficial peroneal nerve territory. A strong Tinel’s sign was elicited over the peroneal tunnel radiating to the lateral compartment of the lower leg.

At 10 months, the patient exhibited some motor recovery in all affected nerves. The FDL and FHL were able to actively flex against gravity and resistance (MRC grade 4). The TP power was found to be MRC grade 3. He displayed active movement of his PL, EDL, and EHL against gravity (MRC grade 3). The TA, however, remained paralysed, although it was noted that this muscle had sustained direct trauma during the index injury. Sensation remained reduced in the superficial peroneal nerve and tibial nerve territories and absent in the deep peroneal nerve distribution.

At 12 months, there were persistently strong Tinel’s signs at the tarsal tunnel and the peroneal tunnel, with increased sensitivity in the anterior scar overlying the superficial peroneal nerve and a strong Tinel’s sign radiating to the dorsum of the foot. After a discussion about his symptoms and the slowing recovery, the patient consented to surgical decompression at the aforementioned sites.

Before the operation, the patient’s VAS score was 60 (1-100). His ten-test score was 0/10 for the deep peroneal nerve territory and 6/10 for the superficial peroneal nerve and tibial nerve sensory distribution. On the day of surgery, the power for PL, EDL, and EHL was MRC grade 3, and for the TA, it was MRC grade 0. At six weeks following surgery, the patient’s VAS score had improved to 40, and the ten-test scores had improved for all three nerves, with the deep peroneal nerve, superficial peroneal nerve, and tibial nerve reported as 1/10, 8/10, and 9/10, respectively. Examination of motor power showed improvement of PL, EDL, and EHL to MRC grade 4 and TA to MRC grade 2.

Case three

A 35-year-old male collided with a tree at high speed while mountain biking. There was axial loading of the spine, a lateral deviation injury to the neck, and depression of the shoulder. He sustained a wedge-compression fracture of the T8 vertebral body plus a traction injury to the brachial plexus with predominant C5 axonopathy, resulting in persistent dysfunction of the suprascapular and axillary nerves. Initially, there was additional loss of the posterior cord with absent triceps function; however, early clinical recovery of the distal posterior cord was seen within two weeks, and neurophysiology confirmed the axonopathy of C5. Over the subsequent six months, the patient experienced recovery of axillary nerve function to deltoid and teres minor, but suprascapular nerve dysfunction was persistent, with wasting of both supraspinatus and infraspinatus muscles. Examination revealed weak shoulder abduction and external rotation consistent with predominant suprascapular nerve dysfunction. The clinical findings were supported by electromyography, which demonstrated axonopathy within the C5 innervated axillary and suprascapular motor territories with poor recruitment and polyphasia in the deltoid muscle, indicative of some reinnervation.

The reinnervation distance to the supraspinatus should support the recovery of this muscle prior to the deltoid in the setting of a C5 injury. An ultrasound was performed to exclude a concomitant rotator cuff tear. In view of the persisting denervation of the supraspinatus and infraspinatus, a posterior decompression of the suprascapular nerve at the suprascapular ligament was advised to determine whether these findings were due to compression of the recovering nerve at the suprascapular notch or due to an avulsion-type injury. This was important to rule out, as the traction of the suprascapular nerve with shoulder depression and contralateral cervical spine flexion may rupture the nerve in the notch where it is tethered by the suprascapular ligament.

Surgery was carried out nine months post-injury. The suprascapular nerve was found to be in continuity with poor stimulation thresholds that improved immediately following decompression. Examination two weeks later revealed a reduction in shoulder abduction strength due to the trapezius muscle splitting posterior approach to the suprascapular notch. This weakness resolved two months post-operatively, with a strong MRC grade 4 shoulder abduction with good recruitment and muscle bulk in both the deltoid and supraspinatus. The external rotation in abduction (teres minor) was a strong MRC grade 4, and the external rotation of the adducted shoulder (infraspinatus) was a weak MRC grade 4. At final review, nine months following surgery, shoulder abduction was MRC grade 5 with a strong external rotation MRC grade 4 in shoulder adduction and abduction. Moreover, both the supraspinatus and infraspinatus muscles had recovered their bulk.

It is reasonable to deliberate about whether the neurolysis performed when initially confirming that the suprascapular nerve was in continuity may have been responsible for the positive outcome. However, the immediate improvement in stimulation thresholds recorded intra-operatively following decompression at the suprascapular ligament suggests that there was a persistent conduction block affecting the recruitment of the reinnervated axons within the nerve.

## Discussion

The literature search identified seven relevant studies, a summary of which can be found in Table 2 [3-9].

**Table 2:** Summary of studies included in the literature review PIN: posterior interosseous nerve; THA: total hip arthroplasty; MRC: Medical Research Council power grading score; SRN: superficial radial nerve; BR: brachioradialis, ECRL: extensor carpi radialis longus; EMG: electromyography

Author, Year	Study design	Cases	Injury	Site of decompression	Outcomes	Conclusion
Johnston et al., 1993 [3]	Pre-clinical animal study	-	-	-	-	This experimental study suggests that if a known area of anatomic narrowing exists distal to a nerve repair site, consideration should be given to its surgical release.
Schoeller et al., 1998 [5]	Case report	2	Radial nerve in the upper arm	PIN at the supinator arch	Improvement of motor function	Thorough clinical follow-up is necessary to detect compression and distinguish between other causes of failed regeneration. Preventative decompression at the time of repair may be beneficial.
Wilson et al., 2018 [8]	Retrospective cohort study	37	Sciatic nerve injury during THA	Common peroneal nerve at the peroneal tunnel	65% of the patients in this study recovered dorsiflexion strength of MRC ≥ 3/5 at the latest follow-up.	Common peroneal nerve decompression at the peroneal tunnel can improve outcomes in patients who have suffered iatrogenic sciatic nerve injuries and who do not show signs of spontaneous recovery.
Żyluk et al., 2018 [4]	Case report	1	Median nerve injury in the distal forearm	Median nerve at the carpal tunnel	Improvement of pain and sensation	The authors speculated whether the nerve trauma had any relationship to the occurrence of the compression syndrome.
Morgan et al., 2020 [9]	Retrospective cohort study	21	Brachial plexopathy	Median nerve at the carpal tunnel (49%) and ulnar nerve at the cubital tunnel (40%)	No specific outcomes were reported for these patients	The authors concluded that simple decompression and external neurolysis at one site bolstered recovery at the other.
Heinzel et al., 2022 [6]	Case report	1	Radial nerve in the upper arm	PIN at the supinator arch and SRN between the BR and ECRL	Improvement of motor function and sensation	The possibility of distal nerve compression following a proximal nerve injury must be kept in mind and surgically addressed in a timely manner.
Makhdom, 2022 [7]	Case report	1	Sciatic nerve injury during THA	Common peroneal nerve at the peroneal tunnel	Improvement in pain and sensation. Dorsiflexion improved from MRC 0/5 to 3/5.	Early distal peroneal nerve decompression after THA can be beneficial in selected patients based on the clinical presentation and EMG findings.

This included, four case reports (patients with three radial nerve injuries all of whom underwent decompression of the posterior interosseous nerve at the supinator arch and one who also underwent decompression of the superficial radial nerve between brachioradialis and extensor carpi radialis longus; one median nerve injury with decompression at the carpal tunnel; and, one sciatic nerve injury with decompression of the common peroneal nerve at the peroneal tunnel) [4-7]; a retrospective study that observed 37 patients who received prophylactic common peroneal nerve decompression at the peroneal tunnel after sustaining iatrogenic sciatic nerve injury [8], and a pre-clinical study carried out on rat models clarifying that an area of subclinical compression distal to the site of nerve injury will adversely affect neuroregeneration [3]. Additionally, in their 2020 study, Morgan and colleagues discussed the effectiveness of neurolysis of the distal brachial plexus in the medial brachial fascial compartment for managing pain and functional decline, noting that almost half of their patients also required decompression at the carpal and/or cubital tunnel due to concurrent symptoms of compression at these sites [9].

The scarcity of literature available highlights the medical community’s limited awareness of this phenomenon. This is likely compounded by an expectation of poor recovery with these injuries, the fact that neurophysiological confirmation of compression in the setting of an axonopathic injury is unreliable, and a general lack of consensus on clinical signs [8]. Therefore, it is likely that there is a high rate of underdiagnosis of this poorly recognised phenomenon.

The three cases presented in this study and those identified in the literature review reveal the need for early detection of distal compression in these injuries. The narrow window in which reinnervation can occur, coupled with a limited rate of regeneration, makes timely decompression of vital importance [4-9]. Diagnosis is only possible with vigilant and repeated clinical examination of the patient’s motor and sensory recovery, along with careful noting of new pain and discomfort [4-9]. The regeneration of a nerve that contains a cutaneous sensory component, such as in cases one and two, can be examined by monitoring the development of a distal Tinel’s sign, the differential Tinel’s sign, or its rate of advancement [8]. An attentive examination is especially important given that neurophysiology cannot accurately reveal the presence of compression where the nerve is already injured, owing to the low amplitudes measured during these studies [16]. Table 3 summarises the indications used in our practice to guide when to consider decompressive surgery following a proximal nerve injury.

**Table 3:** Indications to consider decompressive surgery following a proximal nerve injury

Examination findings
A static Tinel’s point at a known compression point
A weak and slowing distal Tinel’s sign
Persistent neuropathic pain
Direct compression of the suspected entrapment site causing exacerbation of pain and paraesthesia
Poorer-than-expected motor or sensory recovery

The ultimate goal with any peripheral nerve injury is complete functional recovery. Nerve regeneration is a notoriously slow process, with a rate of progression of 3 mm per day following low-grade axonopathy with axonal discontinuity but intact nerve sheath (Sunderland grade 2) in proximal injuries close to the axon cell body. Slower progression is seen in higher-grade injuries (Sunderland grade 3) where there is some disruption of the nerve sheath, and these injuries will have inferior outcomes. A rate of 1 mm per day is typical for a nerve following microsurgical repair. A rate lower than 1 mm per day or a non-progressive Tinel’s sign is an indicator of high-grade injury (Sunderland grade 4), and, usually, there will be a poor outcome with no useful functional recovery [17]. Regeneration is limited by the reinnervation distance, the severity of the nerve injury, the degree of concomitant end-organ damage, and, in cases of nerve transection injury, the delay between injury and repair [1, 17].

Incomplete recovery is common, and the accepted window for motor reinnervation to occur is within 12-18 months, with the potential for sensory reinnervation slightly longer at approximately two years [18]. In proximal injuries, there is limited functional organisation of the nerve, and some partial axon continuity may remain in all distal muscles supplied by the nerve due to interfascicular branching along its course. In such cases, there is the potential for a longer window for successful reinnervation due to the adoption of denervated motor fibres by an intact axon within the intra-muscular neural plexus. This may, in part, explain the late recovery seen in some injuries with nerve sheath continuity [19].

Many patients will not fully recover and often exhibit partial or complete loss of both sensory and motor function, as well as experiencing other debilitating symptoms such as neuropathic pain [1-2]. Distal entrapment of an injured nerve can lead to a reduction, or halting, of its regeneration or failure to restore conduction to regenerated axons due to a persisting segmental conduction block at a point of entrapment. Regenerating axons have a growth cone and transient swelling due to increased axoplasmic transport. The swollen nerve is susceptible to entrapment, which can lead to ischaemia, demyelination, and impaired axoplasmic streaming, rendering a nerve with the potential for function or further regeneration useless. The notion of a ‘double-crush’ syndrome should also be considered. This concept suggests that a proximal subclinical injury to a nerve can predispose it to subsequent lesions in its distal course [20]. This could explain, in part, why, in clinical practice, some individuals are affected more than others.

Limitations

This case series is subject to several limitations. Firstly, it is observational in nature, and the sample size is limited to three cases, which impedes the generalisability of the results. Secondly, the cases presented represent differing patterns of injuries that occurred in three distinct anatomical locations. The heterogeneity of the cases led to the use of a variety of outcome measures, including several subjective tools, and, consequently, any direct comparison between the cases is constrained. However, the heterogeneity of the cases presented may also be interpreted as a strength, as it replicates real-life clinical practice where no two nerve injuries are the same. Additionally, this case series highlights that this issue is pertinent to patients with nerve injuries in both the upper and lower limbs. Furthermore, the study exposes the difficulties encountered in proving the efficacy of decompressive surgery for this particular phenomenon. To account for this, immediate intra-operative findings, such as improved response to stimulation post-decompression, were recorded. Early improvement intra-operatively and at early clinic review would suggest that the improvement is more likely due to the decompression than any nerve recovery that might have happened without intervention.

## Conclusions

Although we report a series of only three cases, these narratives, along with the findings of the studies included in our literature review, indicate that distal entrapment of proximally injured nerves is a recurrent problem faced by nerve surgeons. We propose that targeted peripheral nerve decompression can be an appropriate treatment for patients who display signs of stalled regeneration. This paper adds to the limited discourse on this phenomenon; however, there is a need for further research with greater numbers and using a standardised protocol. This has resulted in the authors working towards developing a prospective observational study of consecutive patients that will investigate the indications, value, predictors of success, and practicality of decompression surgery for this phenomenon.
